# Factors That Contribute to Contraceptive Stockout Rates in Nigerian Health Facilities

**DOI:** 10.1111/sifp.70043

**Published:** 2026-01-21

**Authors:** Taiwo Ibinaiye, Babatunde Adelekan, Ummulkhulthum Bajoga, Sampson Ezikeanyi, Amaka Anene, Ishioma Ntaka‐Okocha, Collins Opiyo, Andat Dasogot, Koessan Kuawu

## Abstract

Contraceptive stockouts are a major barrier to effective family planning (FP) service delivery in Nigeria, limiting access to modern methods and contributing to adverse reproductive health outcomes. Despite ongoing efforts to strengthen the supply chain, many health facilities continue to experience stockouts. A cross‐sectional study was conducted in September 2024 across 1,050 service delivery points (SDPs) in Nigeria. Descriptive statistics and univariate mixed‐effects logistic regression were used to explore associations between stockouts and facility characteristics, including location, supervision frequency, resupply methods, and logistics practices. Overall, 41.7 percent of SDPs reported at least one contraceptive stockout in the three months preceding the survey. Stockouts were slightly more common in rural facilities (56.8 percent) compared to urban facilities (43.2 percent), though this difference was not statistically significant (*p* = 0.53). Monthly supervisory visits were associated with significantly lower stockout rates (*p* = 0.014). Facilities relying on external agencies for resupply had 1.55 times higher odds of stockouts than those calculating needs internally (*p* = 0.058). Delays exceeding two weeks between ordering and delivery were the strongest predictor of stockouts (odds ratio: 1.76, 95 percent confidence interval: 1.257–2.474, *p* < 0.001). Improving supply chain efficiency, supervision frequency, and resupply models is critical to reducing contraceptive stockouts and enhancing FP service delivery in Nigeria.

## INTRODUCTION

Contraceptive stockouts remain a persistent barrier to the delivery of quality family planning (FP) services in Nigeria and across Sub‐Saharan Africa, where unmet need for FP is still high (United Nations Population Fund 2017; National Population Commission and ICF 2019; Adefalu et al. 2019). Stockouts, defined as the unavailability of one or more contraceptive methods at health facilities, undermine individuals’ ability to make informed reproductive choices and contribute to adverse outcomes such as unintended pregnancies and increased maternal mortality (Ministry of Health Uganda 2014; Wang and Mallick 2019).

Despite efforts to improve FP access in Nigeria, many health facilities, particularly in underserved areas, continue to experience frequent stockouts (Iryanyawera 2016; Ministry of Health Uganda 2014; Adefalu et al. 2019; Fagbamigbe, Afolabi, and Idemudia 2018). These disruptions disproportionately affect vulnerable populations and reflect broader challenges on both the demand and supply sides of service delivery. Demand‐side barriers include sociocultural opposition, limited awareness, and economic constraints (Guttmacher Institute 2009; United Nations Department of Economic and Social Affairs 2018; Choge et al. 2021; Ministry of Health Uganda 2014), while supply‐side factors involve inadequate funding, inefficient logistics, and weak data systems (Ezenduka et al. 2014; Ali and Okud 2013; Altijani et al. 2023; Daff et al. 2014). Lessons from countries like Senegal and Haiti underscore the effectiveness of proactive supply chain models and diverse contraceptive options in addressing these issues (Wang and Mallick 2019; Muhoza et al. 2021).

This study provides a cross‐sectional analysis of contraceptive stockouts in Nigerian health facilities, exploring the rate, drivers, and potential solutions. By using quantitative methods, it seeks to offer actionable insights to enhance contraceptive availability and support national reproductive health goals.

## METHODS

The study utilized a cross‐sectional survey design to evaluate both the rate and contributing factors associated with contraceptive stockouts in health facilities across Nigeria. A minimum sample size of 30 facilities was calculated for each of the 36 states based on the assumption that the distribution of sample means will be approximately normal regardless of the population's distribution (ICF 2022). A total of 1,110 facilities were selected from the states through a multistage sampling technique. At the first stage, the number of facilities was proportionally allocated, and then the number of facilities by category (primary, secondary, or tertiary) in each state was calculated and selected based on simple random sampling. Out of 1,100 facilities selected, survey response was completed in 1,050 facilities (95 percent). The data were collected through observations of physical contraceptive inventories and structured survey interviews with key facility staff, including service providers and clients. The survey was organized across six main data collection domains, including general facility information, contraceptive and maternal health supplies, FP supply chain logistics, cold chain capabilities, staff training, and supervision, covering 36 states and the Federal Capital Territory (FCT), Abuja.

### Survey Design and Facility Sampling

The sampling frame was constructed from a list of health facilities offering FP and maternal health services, as provided by the Federal Ministry of Health (FMoH) and State Ministries of Health (SMOH). This list was categorized by facility level (primary, secondary, or tertiary), management type (private or public), and urban or rural location. The sample frame comprised 30,341 primary‐level facilities, 4,018 secondary‐level facilities, and 48 tertiary‐level facilities. A stratified sampling approach was employed, with facilities stratified by their provision of modern contraceptive methods and reproductive health services. This strategy ensured a representative sample, targeting a minimum of 30 facilities per state.

The sample size was calculated based on the minimum sample size expected in each domain and state (ICF 2022), which provides a formula to determine the minimum sample size per domain. The calculation was based on assumptions of normal distribution and incorporated higher sampling probabilities for secondary and tertiary facilities to accommodate their limited representation in the sampling frame.

### Data Collection

Trained data collectors visited the selected health facilities to conduct interviews with the facility in‐charge, FP providers, pharmacy staff, and labor ward providers. The data collection instruments were structured questionnaires designed to capture data on various factors, including general facility information, contraceptive and maternal health supplies, FP supply chain logistics, cold chain capabilities, staff training, supervision, guidelines/protocol availability, use, user fees, waste disposal, and client satisfaction with FP services.

Data were collected using electronic devices (smartphones or tablets) equipped with survey software, allowing for real‐time data entry and validation to enhance accuracy and data quality. Data management procedures involved thorough cleaning, coding, and verification to ensure consistency and reliability before proceeding with analysis. The final dataset for analysis comprised 1,050 facilities regularly providing FP services, of which 610 were primary level, 387 secondary, and 53 tertiary facilities.

### Statistical Analysis

Descriptive and inferential analyses were performed using data derived from both observational assessments and structured interview responses. Observational data included direct verification of contraceptive stock levels and physical inventory checks conducted at each facility, while interview data were obtained from facility in‐charges, FP providers, and pharmacy staff using structured questionnaires. Stockout was categorized as “1” and no stockout as “0.” Categorical variables were summarized as frequencies and percentages. Analysis included determining the rate of contraceptive stockouts by method and evaluating associations between variables. For each method, the stockout rate is calculated as the proportion of facilities reporting a stockout among facilities that routinely offer/are mandated to provide that method; facilities outside a method's scope are excluded from that method‐specific denominator.

Subsequently, univariate multilevel mixed‐effect logistic regression models, incorporating random intercepts for clusters, quantified the association between various factors and contraceptive stockout outcomes. States served as the clustering unit; observations from facilities within the same state were treated as correlated. Odds ratios (OR) and 95 percent confidence intervals (CI) were calculated, with statistical significance set at a *p*‐value of <0.05.

### Ethical approval

Ethical approval for this assessment falls under the broader authorization and mandate granted for the National Family Planning Programme, implemented jointly by the FMoH and the United Nations Population Fund (UNFPA). This approval is covered under the FMoH with reference number MH.8072/Vol.1. All program activities, including the UNFPA Supply Survey, are further authorized under the UNFPA Policies and Procedures Manual and the Standard Co‐Financing Agreement between UNFPA and the FMoH, which provides institutional ethical oversight for routine FP program monitoring activities.

Informed consent was obtained verbally from all participants prior to data collection. Enumerators documented each participant's consent through the electronic data collection system, which automatically recorded the date and time of consent. Participants were informed about the purpose of the assessment, their voluntary participation, their right to withdraw at any point without any consequence, and the confidentiality and anonymity of their responses.

## RESULTS

The analysis of contraceptive stockout rate across Nigerian health facilities provides insight into the availability challenges of various contraceptive methods at service delivery points (SDPs). Out of a total of 1,050 SDPs, 41.71 percent reported experiencing a stockout of at least one contraceptive method, while 58.29 percent had no stockouts, indicating a notable portion of facilities still face stockout issues, which may limit clients' access to their preferred FP methods (see Online Appendix).

**TABLE 1:** Percentage of contraceptive stockouts by SDP factors

	No stockout		Stockout		Total		
	No.	%	No.	%	No.	%	*p*
SDP is located in an urban area or a rural settlement							
Urban	276	45.1	189	43.2	465	44.3	0.53
Rural	336	54.9	249	56.8	585	55.7	
Total	612	100	438	100	1,050	100	
Level of service delivery point							
Primary level care SDPs	347	56.7	263	60.0	610	58.1	0.56
Secondary level care SDPs	233	38.1	154	35.2	387	36.9	
Tertiary level care SDPs	32	5.2	21	4.8	53	5.0	
Total	612	100	438	100	1,050	100	
Management of the service delivery point?							
Government	547	89.4	395	90.2	942	89.7	0.67
Private and others	65	10.6	43	9.8	108	10.3	
Total	612	100	438	100	1,050	100	
What is the distance between the location of the health facility and the nearest location where SDP receives its regular supplies?							
<10 km	270	44.1	173	39.5	443	42.2	0.13
>10 km	342	55.9	265	60.5	607	57.8	
Total	612	100	438	100	1,050	100	
Are there staff working at this SDP who are trained to provide modern contraceptive methods							
Yes	575	94.0	414	94.5	989	94.2	0.70
No	37	6.0	24	5.5	61	5.8	
Total	612	100	438	100	1,050	100	
Number of staff trained to provide modern contraceptives							
No training	37	6.0	24	5.5	61	5.8	0.70
Trained at least one	575	94.0	414	94.5	989	94.2	
Total	612	100	438	100	1,050	100	
When was the last time this facility was visited by a supervisory authority?							
In less than one month	316	51.6	202	46.1	518	49.3	0.078
One month and above	296	48.4	236	53.9	532	50.7	
Total	612	100	438	100	1,050	100	
How frequently does this facility receive visits from the supervisory authority?							
Monthly	395	64.5	250	57.1	645	61.4	0.014
More than months	217	35.5	188	42.9	405	38.6	
Total	612	100	438	100	1,050	100	
Facility has available family planning guidelines							
Yes	525	85.8	352	80.4	877	83.5	0.020
No	87	14.2	86	19.6	173	16.5	
Total	612	100	438	100	1,050	100	
SDP uses any logistics forms for reporting and ordering supplies							
Yes	564	92.2	391	89.3	955	91.0	0.11
No	48	7.8	47	10.7	95	9.0	
Total	612	100	438	100	1,050	100	
Facility uses any form of information communication technologies (ICT) system							
Yes	475	77.6	358	81.7	833	79.3	0.10
No	137	22.4	80	18.3	217	20.7	
Total	612	100	438	100	1,050	100	
How are the resupplies for contraceptives for this facility determined?							
based on the calculation of the quantity needed	558	91.2	376	85.8	934	89	0.007
determined by the institution/warehouse	40	6.5	46	10.5	86	8.2	
Any other ways of resupply are determined	14	2.3	16	3.7	30	2.9	
Total	612	100	438	100	1,050	100	
Main source of your routine medicines and supplies							
Central medical/state store	226	36.9	167	38.1	393	37.4	0.95
Regional/district warehouse or institution	39	6.4	25	5.7	64	6.1	
Local Government Authority (LGA) medical store on the same site	281	45.9	201	45.9	482	45.9	
NGO/donors/private sources	66	10.8	45	10.3	111	10.6	
Total	612	100	438	100	1,050	100	
How long does it take between ordering and receiving products?							
<2 weeks	386	63.1	192	43.8	578	55.0	
>2 weeks	226	36.9	246	56.2	472	45.0	0.001
Total	612	100	438	100	1,050	100	

SOURCE: UNFPA Supplies Survey 2024.

The stockout rate for male condoms was 22.28 percent, showing that nearly a quarter of SDPs struggled to maintain a steady supply of this commonly used contraceptive method. Female condoms experienced a slightly lower stockout rate at 19.46 percent, though this figure still points to challenges in ensuring consistent access to barrier methods. Both rates reveal the need for enhanced planning and inventory management to better meet demand.

Oral contraceptives, at a stockout rate of 16.65 percent, and injectables, at 13.32 percent, were more consistently available compared to other methods. Their relatively lower stockout rates reflect their widespread use and possible prioritization in supply chains. Nonetheless, these figures indicate that supply disruptions still occur and highlight the need for targeted strategies to sustain continuous access to these popular options.

Emergency contraception had one of the highest stockout rates at 34.06 percent, which is especially concerning given the time‐sensitive nature of this method. Limited availability of emergency contraception could significantly impact FP services, as clients rely on its accessibility to prevent unintended pregnancies following unprotected intercourse. This high stockout rate underscores a critical need for improved supply chain management and prioritization of this method.

Long‐acting reversible contraceptives (LARCs), such as implants and intrauterine devices, had stockout rates of 14.7 percent and 12.07 percent, respectively. Although these rates are relatively lower compared to emergency contraception, they still highlight potential logistical challenges in sustaining the availability of LARCs, which are essential for clients seeking long‐term contraceptive solutions.

Sterilization methods had the lowest stockout rates, with female sterilization at 9.18 percent and male sterilization at 16.13 percent. The lower stockout rates for sterilization may reflect stable demand and possibly fewer fluctuations in inventory needs. However, the slightly higher stockout rate for male sterilization compared to female sterilization suggests possible gaps in supply chain coordination and access to male‐specific services.

The most significant factor affecting stockouts was the time taken between ordering and receiving contraceptive supplies. Facilities that waited more than two weeks for resupply had significantly higher odds of experiencing stockouts compared to those that received supplies within two weeks (OR: 1.764; 95% CI: 1.257–2.474; p < 0.001) (see Table 2). This highlights the critical role of timely resupply in preventing contraceptive shortages.

**TABLE 2:** Results of univariate mixed‐effects logistic regression models of rate and contributing factors to contraceptive stockouts in Nigerian health facilities

Variable	Odds ratio	95% CI		*p*‐value
Frequency of facility receiving visits from supervisory authorities				
Monthly	Ref			
More than months	1.122	0. 825	1.525	0.461
Facility has available family planning guidelines.				
Yes	Ref			
No	1.303	0.852	1.990	0.221
How are the resupplies for contraceptives for this facility determined				
Based on the calculation of the quantity needed	Ref			
Determined by the institution/warehouse/other	1.552	0.985	2.446	0.058
How long does it take between ordering and receiving products?				
<2 weeks	Ref			
>2 weeks	1.764	1.257	2.474	<0.000

## DISCUSSION

The findings of this study highlight the persistent challenge of contraceptive stockouts in Nigerian health facilities and align with previous research on stockout determinants in Sub‐Saharan Africa. Despite efforts to enhance FP service delivery, significant gaps remain, particularly in supply chain management, supervision, and resupply efficiency.

This study found that 41.71 percent of SDPs experienced at least one contraceptive stockout in the last three months before the survey, which is consistent with previous research in Uganda and Rwanda, where stockout rates ranged between 30 percent and 50 percent depending on facility type and location (Adefalu et al. 2019; (Altijani et al. 2023). Similarly, studies from Kenya and Tanzania have documented frequent stockouts of contraceptive methods, particularly in rural and resource‐limited settings (Ali and Okud 2013; Daff et al. 2014). The high stockout rate of emergency contraception (34.06 percent) in this study is particularly concerning, as it aligns with findings from Haiti, where an inconsistent supply of emergency contraceptives was identified as a major barrier to effective FP service delivery (Ministry of Health Uganda 2014). Given the time‐sensitive nature of emergency contraception, addressing these stockouts is crucial to reducing unintended pregnancies.

In Nigeria, this challenge is further compounded by the fact that emergency contraceptives are not included in the Basket Fund (The National Contraceptive Basket Fund is a pooled financing mechanism where multiple donors combine resources into one managed pot to support FP commodity procurement) for contraceptive procurement. As a result, access to emergency contraception remains highly unreliable, particularly in underserved areas. Given the time‐sensitive nature of emergency contraception, addressing these stockouts through increased funding and improved supply chain mechanisms is a crucial option for an individual to prevent pregnancy.

The findings also indicate that stockout rates were slightly higher in rural SDPs (56.8 percent) (see Table 1), though this difference was not statistically significant. This aligns with studies in Ethiopia and Senegal, which found that rural facilities are more vulnerable to supply chain disruptions due to longer distances from supply sources and weaker logistical support (Adefalu et al. 2019; Muhoza et al. 2021). However, our results suggest that urban facilities are not immune to stockouts, reinforcing the need for systemic improvements in supply chain efficiency across all locations.

Supervision emerged as a key factor in stockout prevention. Facilities that received monthly supervisory visits had significantly lower stockout rates than those supervised less frequently (*p* = 0.014) (see Table 1). This aligns with findings from Senegal and Uganda, where frequent supervision improved stock management and reduced supply disruptions (Choge et al. 2021; Gelagay et al. 2023). Condoms serve a dual purpose: they are a FP method (for pregnancy prevention) and a critical tool for Human Immunodeficiency virus/Sexually transmitted infection (HIV/STI) prevention. This dual utility, however, has led to a fragmented supply chain, which contributes to the very stockouts that seem so counterintuitive. In Nigeria, like many countries, health supply chains are often vertical, meaning they are managed separately for specific disease areas. For instance, HIV/STI programs are funded largely by international donors like the Global Fund and USAID (through projects like Global Health Supply Chain Program – Procurement and Supply Management (GHSC‐PSM)). These programs have traditionally had dedicated, well‐resourced, and often more robust supply chains. Their primary goal is to ensure a continuous supply of antiretrovirals, test kits, and condoms to achieve ambitious targets like the UNAIDS 95‐95‐95 goals. While FP systems are often managed by a different set of government agencies and organizations (like UNFPA, GHSC PSM, and Marie Stopes International (MSI)), and other donors procure condoms and utilize a separate supply chain often. Dedicated HIV/STI supply chains often have lower and more consistent stockout rates for condoms compared to FP/Reproductive Health (RH) systems (Sama et al. 2020; Akinyemi et al. 2021). The FP supply chain system is for procuring an adequate method mix of commodities based on the National Quantification exercise done by various stakeholders and led by the Department of Family Health and the Federal Ministry of Health and Social Welfare. The high stockout rate for condoms in the FP system is a critical symptom of an underresourced supply chain.

The time taken between ordering and receiving contraceptive supplies also had a strong influence on stockouts. Facilities that waited more than two weeks for delivery had significantly higher odds of stockouts (OR: 1.764, *p* < 0.001), supporting previous research that highlights delays in procurement as a major bottleneck in contraceptive availability (Fagbamigbe, Afolabi, and Idemudia 2018; Onoja et al. 2020). Similar findings have been reported in Rwanda and Nigeria, where delayed shipments and inefficient inventory management contributed to frequent contraceptive shortages (Ezenduka et al. 2014; Kana et al. 2016). Addressing these delays through improved logistics coordination and demand forecasting could enhance contraceptive security.

Overall, the analysis reveals significant variability in stockout rates by contraceptive method, underscoring the need for a multifaceted approach to strengthen contraceptive supply chains in Nigerian health facilities. Strategies to address these stockouts may include enhanced demand forecasting, more robust logistics, and targeted funding, which can contribute to achieving Nigeria's FP and reproductive health goals by ensuring consistent contraceptive availability.

### Limitations and Areas for Further Research

This study has limitations. First, the analysis relies on self‐reported facility practices and supervisory routines alongside inventory checks, which may be prone to specific reporting biases such as recall bias (e.g., timing and frequency of stockouts within the prior three months), social desirability bias (over‐reporting guideline availability, supervision frequency, or “best‐practice” resupply methods), and misclassification bias (classifying a method as “stockedout” versus “not routinely offered”). Second, although facilities were sampled across all states and the FCT, heterogeneity in record‐keeping quality and regional supply dynamics may limit generalizability. Finally, the regression models were primarily univariate; residual confounding from unmeasured system‐level factors (e.g., upstream warehousing constraints) is possible.

Future studies should evaluate targeted interventions, such as electronic inventory systems and mobile supply‐chain solutions using robust designs: longitudinal/panel studies that link routine Logistic Management information system/District Health Information Software 2 (LMIS/DHIS2) data with facility audits to capture seasonality and lead‐time variability; multivariable mixed‐effects models to account for facility, district, and state‐level clustering; and quasi‐experimental approaches (e.g., pre–post or stepped‐wedge trials) to test supply‐chain reforms like informed‐push or vendor‐managed resupply. Additionally, regional disparities and inconsistent data affect the generalizability of the findings across Nigeria. Future research should adopt a longitudinal design to monitor stockout trends over time and employ mixed method approaches to capture quantitative and qualitative insights from facility staff, clients, and supply chain managers. Further studies could examine the impact of specific interventions, such as electronic inventory systems and mobile supply chain solutions, on stockout reduction.

## CONCLUSION

This study confirms that contraceptive stockouts in Nigerian health facilities are influenced by multiple interrelated factors, including supervision frequency, supply determination methods, resupply timing, and supply chain efficiency. The findings align with broader literature from Sub‐Saharan Africa, reinforcing the need for improved logistics management, more frequent supervisory visits, and the adoption of evidence‐based resupply models such as the informed push system.

Policy interventions should focus on strengthening supply chain infrastructure, reducing procurement delays, and enhancing supervisory mechanisms to ensure timely resupply. Leveraging digital logistics tools is a critical strategy for improving supply chain management in the health sector, particularly in countries with fragmented systems like Nigeria. These tools enhance visibility, data quality, and efficiency, which are all essential for reducing commodity stockouts. Such tools include logistic management information software platforms that manage the flow of information and health commodities across the healthcare system or mobile‐based data collection and reporting applications, often on smartphones or tablets, that allow health workers at the last mile (clinics and community health posts) to report on stock levels and consumption (VillageReach 2023). By addressing these challenges, Nigeria can make significant strides toward ensuring consistent contraceptive availability, thereby supporting broader reproductive health goals and reducing unmet FP needs.

## AUTHOR CONTRIBUTION

TI acted as lead author and was responsible for conceptualization of the study, data analysis, interpretation, report writing, development of the first draft of the paper, and finalization of the manuscript. BA and UB reviewed and approved the study protocol, data analysis, and interpretation, and contributed to critical revision for intellectual content. AA, SE, JC, IN, and ME contributed to the conceptualization of the study, data acquisition, data analysis and interpretation, and manuscript review. CO, AD, and KK contributed to the conceptualization of the study, reviewed and approved the study protocol, and contributed to critical revision for intellectual content. All the authors read and approved the final version of the manuscript before submission.

## CONFLICTS OF INTEREST STATEMENT

The authors declare no conflicts of interest.

## ETHICAL STATEMENT

The ethical approval for this assessment falls under the broader authorization and mandate granted for the National Family Planning Programme, implemented jointly by the Federal Ministry of Health (FMoH) and the United Nations Population Fund (UNFPA). This approval is covered under the FMoH with reference number MH.8072/Vol.1. All program activities, including the UNFPA Supply Survey, are further authorized under the UNFPA Policies and Procedures Manual and the Standard Co‐Financing Agreement between UNFPA and the FMoH, which provides institutional ethical oversight for routine FP program monitoring activities.

Enumerators documented the verbal consent by recording participants’ agreement in the data collection software, which also logged the date and time of consent for each respondent. The participants were informed about the purpose of the study, the voluntary nature of their participation, their right to withdraw at any point without consequences, and the confidentiality of their responses.

## Supporting information



Supporting Information

## Data Availability

The data supporting the findings of this study are maintained in the UNFPA database. Access to the dataset can be granted upon reasonable request. For inquiries regarding data access, please contact Nigeria.rep@unfpa.org.

## References

[sifp70043-bib-0300] Adefalu Adewole A, Ladipo Oladapo A, Akinyemi Oluwaseun O, et al . 2019. “Qualitative Exploration of factors affecting Uptake and Demand for Contraception and other Family Planning Services in North‐West Nigeria.” African Journal Reproductive Health 23(4): 63–74. 10.29063/ajrh2019/v23i4.8. 32227741

[sifp70043-bib-0001] Akinyemi, Olufunmilayo , Amina Abubakar , Funmilayo Oladipo , et al. 2021. “Integration of Family Planning Services into HIV Services in Nigeria: Evidence from the Performance Monitoring and Accountability 2020 Survey in Seven States.” PMA Data. 10.2989/16085906.2021.1925312 34264164

[sifp70043-bib-0002] Ali, Abdel Aziem A. , and Azza Okud . 2013. “Factors Affecting Unmet Need for Family Planning in Eastern Sudan.” BMC Public Health 13 (1): 102. 10.1186/1471-2458-13-102. 23379387 PMC3566931

[sifp70043-bib-0003] Altijani, Nour , Safa M. Eldin , Loulou Kobeissi , et al. 2023. “Trends in Birth Attendants in Sudan Using Three Consecutive Household Surveys (2006–2014).” Frontiers in Global Women's Health 4: 1012676. 10.3389/fgwh.2023.1012676 PMC10498120 37711966

[sifp70043-bib-0004] Choge, Mary , Kenneth Ngure , Enock Echoka , and Timothy Abuya . 2021. “A Comparative Analysis of the Availability of Family Planning Services in Social Franchise and Non‐Franchise Private Health Facilities in Kajiado County, Kenya.” Pan African Medical Journal 38: 380. 34367459 10.11604/pamj.2021.38.380.24055 PMC8308919

[sifp70043-bib-0005] Daff, Bocar M. , Cheikh Seck , Hanane Belkhayat , and Paul Sutton . 2014. “Informed Push Distribution of Contraceptives in Senegal Reduces Stockouts and Improves Quality of Family Planning Services.” Global Health: Science and Practice 2 (2): 245–252. 25276582 10.9745/GHSP-D-13-00171 PMC4168620

[sifp70043-bib-0006] Ezenduka, Charles C. , Brian O. Ogbonna , Obinna I. Ekwunife , Matthew J. Okonta , and Charles O. Esimone . 2014. “Drugs Use Pattern for Uncomplicated Malaria in Medicine Retail Outlets in Enugu Urban, Southeast Nigeria: Implications for Malaria Treatment Policy.” Malaria Journal 13: 243. 24961280 10.1186/1475-2875-13-243 PMC4079827

[sifp70043-bib-0007] Fagbamigbe, Adeniyi Francis , Rotimi Felix Afolabi , and Erhabor Sunday Idemudia . 2018. “Demand and Unmet Needs of Contraception among Sexually Active In‐Union Women in Nigeria: Distribution, Associated Characteristics, Barriers, and Program Implications.” SAGE Open 8 (1): 2158244017754023.

[sifp70043-bib-0008] Gelagay, Abayneh A. , Tigist Andargie , Meklit B. Assefa , et al. 2023. “Magnitude of Unmet Need for Family Planning and Associated Factors among Women in the Extended Postpartum Period in Dabat District, Northwest Ethiopia.” BMC Public Health 23 (1): 1123. 37308903 10.1186/s12889-023-16046-3 PMC10262530

[sifp70043-bib-0009] Guttmacher Institute . 2009. “Adding It Up: The Costs and BFamily Planning and Maternal and Newborn Health.” New York: Guttmacher Institute.

[sifp70043-bib-0011] ICF . 2022. Service Provision Assessment (SPA) Survey Design Manual Rockville, MD: ICF.

[sifp70043-bib-0012] Iryanyawera, Marie Claire . 2016. “Associated Factors with Stock Out for Modern Contraceptives in Health Facilities in Rwanda.” Master's thesis, University of Rwanda.

[sifp70043-bib-0014] Kana, Malam A. , Hauwa Idris , Maryam Abdullahi , et al. 2016. “Prevalence and Determinants of Contraceptive Use in Rural Northeastern Nigeria.” Annals of Nigerian Medicine 10 (1): 3–9.

[sifp70043-bib-0015] Ministry of Health Uganda. 2014. “Facility Assessment for Reproductive Health Commodities and Services.” Kampala: Ministry of Health Uganda.

[sifp70043-bib-0301] Muhoza Pierre, Koffi K Alain, Anglewicz Philip, et al . 2021 “Modern contraceptive availability and stockouts: a multi-country analysis of trends in supply and consumption.” Health Policy and Planning 36 (3): 273–287. 10.1093/heapol/czaa197. 33454786 PMC8058948

[sifp70043-bib-0016] National Population Commission (NPC) [Nigeria] and ICF . 2019. Nigeria “Demographic and Health Survey 2018.” Abuja: National Population Commission [Nigeria] and ICF.

[sifp70043-bib-0017] Onoja, Ali Johnson , Felix Olaniyi Sanni , Paul Olaiya Abiodun , Chris Olusola Ogedengbe , Sheila Iye Onoja , and Aisha Abu . 2020. “Quality of Family Planning Services and Client Satisfaction in Selected Private and Public Health Facilities in Nigeria.” Journal of Middle East and North Africa Sciences 6 (9): 25–35.

[sifp70043-bib-0018] Rotimi, Kehinde. 2024. “Review of Public Health Commodity Distribution Models in Nigeria.” Future Business Journal 10: 48.

[sifp70043-bib-0019] Sama, Samuel , Adewale Fagbamigbe , Yetunde O. Oyebamiji , et al. 2020. “Health Facility Readiness to Provide Family Planning Services in Nigeria.” International Journal of Environmental Research and Public Health 17 (19): 7164.33007908

[sifp70043-bib-0022] United Nations, Department of Economic and Social Affairs . 2018. World Urbanization Prospects: The 2018 Revision.

[sifp70043-bib-0023] United Nations Population Fund (UNFPA) . 2017. Family Planning. New York: UNFPA.

[sifp70043-bib-0024] USAID | DELIVER PROJECT . 2011. “Logistics Indicators Assessment Tool (LIAT).” Washington, DC: USAID.

[sifp70043-bib-0025] USAID | DELIVER Project . 2009. “Logistics Management Information System.” Washington, DC: USAID.

[sifp70043-bib-0026] VillageReach . 2023. “Innovations in Digitizing Health Supply Chains in Africa.” Seattle: VillageReach.

[sifp70043-bib-0027] Wang, Wenjuan , and Lindsay Mallick . 2019. “Family Planning Method Choices and Modern Contraceptive Use in Haiti.” BMJ Global Health 4 (Suppl 5): e000765. 10.1136/bmjgh-2018-000765 PMC6606068 31321089

