# Supplementary material for: Factors That Contribute to Contraceptive Stockout Rates in Nigerian Health Facilities
Source: Stud Fam Plann. 2026 Jan 21;57(1):110–21. doi: 10.1111/sifp.70043 (PMC12996751; doi:10.1111/sifp.70043)
Supplement: Supplementary file 1 — Supporting Information [file SIFP-57-110-s001.docx]

Annex A: Percentage of stockout by contraceptive method in the SDP that regularly provide them

| Contraceptive methods | N | Percent |
| --- | --- | --- |
| Stockout status |  |  |
| stock out | 438 | 41.71 |
| no stock out | 612 | 58.29 |
| Total | 1050 |  |
| Male condoms |  |  |
| stock out | 219 | 22.28 |
| no stock out | 764 | 77.72 |
| Total | 983 |  |
| Female Condoms |  |  |
| stock out | 166 | 19.46 |
| no stock out | 687 | 80.54 |
| Total | 853 |  |
| Oral Contraception |  |  |
| stock out | 166 | 16.65 |
| no stock out | 831 | 83.35 |
| Total | 997 |  |
| Injectables |  |  |
| stock out | 136 | 13.32 |
| no stock out | 885 | 86.68 |
| Total | 1021 |  |
| Emergency contraception |  |  |
| stock out | 157 | 34.06 |
| no stock out | 304 | 65.94 |
| Total | 461 |  |
| IUDs |  |  |
| stock out | 102 | 12.07 |
| no stock out | 743 | 87.93 |
| Total | 845 |  |
| Implants |  |  |
| stock out | 142 | 14.70 |
| no stock out | 824 | 85.30 |
| Total | 966 |  |
| Sterilisation for Females |  |  |
| stock out | 19 | 9.18 |
| no stock out | 188 | 90.82 |
| Total | 207 |  |
| Sterilisation for Male |  |  |
| stock out | 20 | 16.13 |
| no stock out | 104 | 83.87 |
| Total | 124 |  |
